# Signal-aware deep learning–based respiratory motion prediction for lung tumor management

**DOI:** 10.3389/fonc.2026.1735140

**Published:** 2026-02-13

**Authors:** Kaushik Pratim Das, Chandra J., Partha Pratim Medhi

**Affiliations:** 1 Department of Computer Science, School of Sciences, Christ University, Bangalore, Karnataka, India; 2 Department of Radiation Oncology, All India Institute of Medical Sciences, Guwahati, Assam, India

**Keywords:** artificial intelligence, lung cancer, radiotherapy, respiratory motion, treatment planning

## Abstract

**Introduction:**

Respiratory motion management in radiotherapy for lung cancer patients remains a significant challenge, as it directly affects accurate tumor targeting. Furthermore, unaccounted tumor motion during treatment planning and delivery can lead to imaging artifacts and biased dose distributions, which compromises the accuracy of image-guided radiotherapy. This issue places clinicians in a dilemma between expanding treatment margins, which increases radiation exposure to healthy tissue or risking reduced targeting precision.

**Methods:**

In this work, a hybrid deep learning model composed of dilated convolutional layers, bidirectional long-short term memory layers, and a generative autoencoder module is proposed to jointly model the spatial and temporal characteristics of respiratory motion, while enabling reconstruction of the physiologically coherent respiratory signals. Each architectural component learns complementary motion-related patterns from respiratory signals to support tumor motion prediction. The model performs motion-range classification, captures abnormal breathing patterns across spatial and temporal domains, reconstructs physiologically coherent respiratory cycles, and predicts tumor motion within an algorithmic validation framework.

**Results:**

Experimental evaluation demonstrates high motion-range classification performance of 98.37%, including low root-mean square error in motion prediction, while maintaining stable performance across long and complex respiratory signals over multiple breathing cycles.

**Discussion:**

This study focuses on algorithmic feasibility and establishes a computational foundation for future clinically calibrated and dosimetrically validated models. The findings indicate that the proposed approach can support future motion-aware radiotherapy planning strategies by improving motion characterization at the algorithmic level.

## 1 Introduction

Lung cancer poses a significant challenge to global public health, exhibiting a substantial mortality rate. According to the World Health Organization (WHO), lung cancer stands as the foremost cause of high mortality, representing approximately 1.8 million (18%) of cancer-related deaths in 2020 (1). These alarming statistics highlight the urgent need for increased awareness, proactive measures, and the development of efficacious treatment options to combat lung cancer effectively. Radiation therapy serves as a viable targeted treatment approach for cases where patients have localized tumors or are unsuitable for surgical resections. In addition, radiotherapy is often considered when patients do not respond to surgery or chemotherapy (2, 3). The utilization of radiotherapy is vital in effectively managing the treatment needs of cancer patients as it offers both curative and palliative benefits. As a standalone treatment method, radiotherapy has demonstrated its capability to achieve remission in approximately 40% of cancer cases (4). Recent advancements in the field of oncology have led to the introduction of advanced techniques like image-guided radiotherapy (IGRT) for treating lung cancer. The primary interaction between technology and the patient occurs in three main stages of the radiotherapy process: medical imaging, treatment planning, and delivery. The most basic form of IGRT involves patient alignment, which is necessary to update the original image utilized for planning with the most recent set of patient images. While the fundamental processes remain unchanged, the time and frequency of these processes are altered. There is a necessity to repeat these procedures during treatment planning, which places significant demands on time and resources. Therefore, there is a need for solutions to automate tasks like segmentation, image registration, and, most importantly, adaptive and real-time replanning (5).

The IGRT approach integrates various imaging modalities, including Positron Emission Tomography (PET) and Computed Tomography (CT), enhancing targeting accuracy in 25% to 50% of cases. The fusion of PET and CT images indicates whether adjustments to the patient’s position are necessary to prevent excessive radiation to healthy tissues (6). Furthermore, using Four-Dimensional Computed Tomography (4DCT) enables personalized radiotherapy by accommodating tumor motion (7). In addition, adapting treatments based on anatomical information from CT and incorporating functional imaging modalities like PET can enhance radiotherapy outcomes (8). However, the primary causes of local failure include three key factors: geometric inaccuracies resulting from limitations in imaging tools utilized for staging and radiotherapy planning, geometric inaccuracies arising from respiratory-induced tumor motion during radiation administration, and insufficient dosage due to concerns regarding potential toxicity (9). Integrating PET/CT in radiotherapy planning and accommodating individualized tumor motion using 4DCT offers a promising approach to enhance tumor targeting accuracy and minimize adverse effects (10, 11).

4DCT is implemented to address respiratory motion artifacts, wherein a gating signal triggers the scanner at specific phases or amplitudes (gates or bins) of the motion cycle. The scanner partitions the patient’s respiratory cycle into a series of phases or amplitude ranges and organizes the data accordingly into these segments. Consequently, images corresponding to different phase or amplitude ranges can be reconstructed from their respective datasets (12). Although 4DCT phase binning is commonly utilized, this approach proves less effective in irregular breathing patterns. Moreover, data obtained through this method may require increased precision due to variations in slopes, periods, and amplitudes. An alternative method is 4DCT amplitude binning, but it does not differentiate between the duration of time spent at the same amplitude level. This approach may result in the omission of images if amplitude values at the end-inhalation and exhalation phases are not attained (13, 14). In addition, 4DCT cannot effectively manage irregular breathing patterns, resulting in misaligned images due to amplitude fluctuations (15). While 4DCT addresses certain aspects of respiratory motion, it suffers from reduced temporal resolution when using phase-sorting techniques, leading to less refined images (16).

Radiotherapy also uses respiratory gating, which involves tumor irradiation at a specific stage of the respiratory cycle, most commonly at the expiration (17). The existing respiratory gating systems are characterized by a time period limit of 3 seconds and an amplitude limit of 4 millimeters (mm), and alternative clinical options should be explored for patients with a breathing period of less than 3 seconds or an amplitude of less than 4 mm. The findings of this study highlight that respiratory gating systems are incapable of gating due to limitations in the time period (>3s) or amplitude (>4mm). Under extraordinary circumstances featuring a time frame of 1 or 2 seconds or an amplitude of 2 or 3 mm, the recording of respiratory curves becomes unattainable. Moreover, there are challenges associated with gating some patients due to shortness of breath during breathing cycles or the presence of small amplitudes (<4mm) (18). Moreover, the efficacy of gating may differ among patients, with the precision and dosimetric advantages of gating reliant on the dependability of the gating device in detecting target motion (19).

In radiotherapy, the breath-hold delivery technique is utilized to restrict respiratory tumor movement within defined volumes. However, this method is challenging for lung cancer patients with pre-existing pulmonary conditions and compromised lung function (20, 21). These patients present the lowest compliance levels and are least capable of adhering to breath-hold techniques. In addition, their breathing patterns are irregular during radiation delivery (7). During gated radiotherapy, the setup phase necessitates monitoring the fiducial and breathing signals to establish the patient’s position and set the gating window threshold. Presently, there is an absence of integrated functionality and a lack of universally applicable methods for most conventional linear accelerators. Therefore, the user manually performs this procedure. Consequently, the accuracy of patient setup and treatment delivery depends on the user’s proficiency (22).

Multileaf collimator (MLC) systems are essential components of modern radiation therapy machines, including linear accelerators, commonly used for IGRT procedures. MLC systems allow for the precise shaping of radiation beams to conform to the specific dimensions and contours of the tumor and the surrounding critical structures during the image-guidance process (23). Therefore, MLC systems play a crucial role in IGRT by enabling accurate radiation delivery to the target while minimizing exposure to healthy tissues. However, there is inherent latency in deploying the MLC system for treatment delivery, which can lead to geometric errors. These delays occur during the acquisition and analysis of respiratory information, which is critical for determining leaf positions and the precise execution of treatment (24).

Effectively addressing motion is paramount in radiotherapy because respiratory-induced tumor movement significantly contributes to patient anatomy errors and tumor localization accuracy. These errors can arise during the planning and radiotherapy treatment delivery phases. A potential solution is introducing a temporal dimension into planning and treatment procedures to mitigate these challenges (25). The necessity of integrating temporal information highlights the significance of respiratory signals in achieving accuracy in predicting tumor motion variations at each time interval. These advanced models can accommodate irregular breathing patterns and consider system latency factors to provide accurate information about the tumor motion range and mitigate potential errors in radiotherapy treatment planning and delivery.

The proposed research presents a hybrid deep generative network designed to address the shortcomings of conventional radiotherapy motion-management techniques by providing a data-driven approach to respiratory motion prediction. The primary contributions are as follows:

1. The model effectively handles respiratory signals, including those with irregular breathing patterns, to enable the identification of various motion ranges within each breathing cycle.
2. The proposed model demonstrates predictive capabilities for tumor motion across time horizons of 50 to 500 milliseconds, which highlights its ability to capture both short and medium-term respiratory dynamics.
3. Experimental results indicates that the model maintains high predictive accuracy across increasing time delays and data volumes, while consistently achieving low root-mean-square-error (RMSE) values even when predicting up to 500 milliseconds ahead, thereby minimizing motion-prediction errors.
4. The hybrid model effectively manages extended signal variations and reconstructs irregularities to generate physiologically consistent breathing curves over time.
5. The model also predicts excessive motion amplitudes, which can provide insights that can support clinicians in understanding respiratory-induced variability relevant to treatment planning.
6. The unique strength of the hybrid deep generative model lies in its multifunctional approach, encompassing four essential tasks: (i) motion-range classification, (ii) tumor-motion prediction, (iii) reconstruction of irregular respiratory signals through accurate breathing-curve generation, and (iv) detection of motion amplitudes exceeding nominal clinical thresholds.

While the proposed model demonstrates high predictive accuracy using respiratory-signal data, the current study focuses on algorithmic validation. Future extensions will incorporate physical-space calibration (in millimeters) and dosimetric evaluation to directly quantify clinical impact. As such, this study provides the computational groundwork necessary for developing subsequent models with enhanced clinical interpretability.

### 1.1 Related works

A significant challenge in treating lung tumors is respiratory-induced pulmonary movement, which can potentially cause displacement of the target of interest and damage surrounding healthy tissues (26). Therefore, adapting a treatment plan responding to any observed tumor motion is critical. One potential method for monitoring tumor positional variations over time involves incorporating a temporal dimension into conventional three-dimensional computer tomography (3D CT) or utilizing 4DCT for treatment planning. In this context, continuous acquisition of CT images throughout the respiratory cycle is essential for ascertaining the potential position of the tumor at any given time within the patient’s breathing cycle (27).

The treatment planning phase is another substantial challenge within the radiation therapy treatment chain. The current treatment planning method primarily depends on manual, time-consuming processes. Manual intervention necessitates planners to adjust and determine plan optimization parameters, making them heavily reliant on the planner’s expertise. In addition, balancing tumor control and potential organ complications is labor-intensive, which may require hours or days to complete a single case. This method involves trial and error, numerous iterations, and significant human involvement. Therefore, artificial intelligence (AI) enabled automated treatment planning models have recently been proposed to streamline the process and ensure consistency and high-quality treatment outcomes (28).

The emergence of tumor motion prediction models offers promising solutions to tackle various challenges in estimating pulmonary tumor positions throughout a respiratory cycle (29). Numerous studies have highlighted the potential of the machine and deep learning models to enhance radiotherapy by providing critical details, such as tumor shape identification, predicting positional changes over time, baseline shifts, toxicity and risk modeling, tumor boundary segmentation and contouring, and lung movement prediction. These models are crucial in minimizing geometric errors in radiotherapy treatment, leading to precision oncology (30–32). AI models have been proposed in radiotherapy as a solution for various challenges. Recently, convolutional long-short-term memory networks (LSTM) have been proposed to predict future frames in the video mode of 4DCT, considering pulmonary movement during breathing cycles (33). While this method generated satisfactory outcomes for precise margin delineation, it is critical to note that LSTM models encounter several issues associated with high computational complexity (34).

In a separate study, online and offline LSTM networks were reported for predicting respiratory motion. The study highlighted the efficacy of LSTM models in predicting up to 500 milliseconds (ms) ahead, resulting in an RMSE of 1.20 mm and 1.00 mm. The authors also stressed the potential for considerable performance variations across different patients. Furthermore, the findings indicate that LSTM models can accommodate latencies observed in MLC tracking systems (35). To develop an accurate and generalized model for predicting respiratory signals that encompass diverse breathing patterns, researchers are also investigating the potential of LSTM networks. In this context, a study leverages data acquired from real-time position management (RPM) systems with superior optimization of LSTM hyperparameters using an exhaustive grid search and by examining factors like the number of layers, hidden units, optimizer, learning rate, epoch and time lags, the optimized LSTM outperformed conventional artificial neural network (ANN) concerning accuracy, leading to significantly reduced errors. However, the study emphasizes the importance of hyper-parameter tuning in LSTM models for respiratory signal prediction (36). While these LSMT-based approaches demonstrate strong temporal modeling capability, they typically operate either as standalone predictors or require extensive hyperparameter tuning, and they do not explicitly incorporate signal reconstruction mechanisms to regularize irregular respiratory patterns.

Another study focused on a multi-scale convolutional neural network using Empirical Decomposition (EMD). The primary objective of this model was to predict respiratory motion over different time intervals. The initial sequence undergoes decomposition using EMD, after which a depth prediction model is applied. The performance evaluation involved a comparison of prediction accuracy and efficiency at different levels. Nevertheless, the authors acknowledge certain limitations, particularly the need to establish a strong correlation between external respiratory signals and the internal motion of tumors (37). Concerning respiratory motion prediction in scenarios with extended latency periods, a deep-bidirectional LSTM model was employed in a recent study. This model could predict respiratory motion with a latency of approximately 400 milliseconds. The authors reported achieving a RMSE of 0.097 mm. However, the model was trained on a relatively small dataset, indicating the potential for enhancement by evaluating the performance on a more extensive and diverse dataset (38).

In addition, convolutional neural networks (CNN) have also been proposed for respiratory motion prediction. The study implemented a CNN in the temporal domain, utilizing external surrogate signals to predict internal target positions. Based on the findings, it can be inferred that the proposed model exhibits submillimeter precision in predicting respiratory signals. The RMSE values recorded were 0.49 mm, 0.28mm, 0.25mm, and 0.67 mm in three dimensions (39). Machine learning (ML) models have also been developed to predict the relationship between external and internal factors in lung tumor motion. These models utilize radiomic features extracted from 4DCT images. The results suggest that ML models offer high sensitivity and specificity, which indicate their efficiency in predicting respiratory motion correlation and extracting characteristics of tumor movement (40). In another study, an ML-based respiratory motion model was developed to accommodate extreme respiratory conditions for percutaneous puncture interventions. The model leverages principal component analysis (PCA) and support vector regression (SVR) to establish the framework. A novel data augmentation method is proposed to augment model robustness and response capacity under extreme conditions. The evaluation demonstrates superior motion prediction and accuracy while outperforming the reference model (41). These methods highlight the effectiveness of learning-based models for respiratory motion prediction. However, they often rely on separate preprocessing or surrogate correlation stages rather than integrating feature extraction, temporal modeling, and signal regularization within a unified framework.

The advancement of radiotherapy systems also necessitates accurate respiratory motion prediction to enable tumor targeting, particularly in the thorax and upper abdomen, where precise dose delivery is crucial. However, a diverse range of breathing patterns can challenge explicit models. To address the challenge, CNN models have been explored to achieve diverse outcomes, such as procedural clustering based on multiple patient breathing patterns and intra-procedural prediction and correlation using both CNN and Kalman filters. In addition, the comparative evaluation indicates that such models can outperform Recurrent Neural Networks (RNN) with a substantial performance improvement based on normalized RMSE (42). While most studies focus on intra-fractional variation within a single treatment session, the inconsistent inter-fractional variation between radiotherapy sessions poses a largely unaddressed and unpredictable challenge.

Furthermore, reducing computational time for predictions is crucial. To address these challenges, a recent study proposed a novel predictor called intra and inter-fraction fuzzy deep learning (IIFDL), which integrates breathing clusters and enhances precision while minimizing computational necessities. The experimental results demonstrate improvement in prediction accuracy compared to existing methods (43). Recent research compared the Gated Recurrent Unit (GRU) with ANNs to create a prediction model for external beam radiotherapy. The GRU model demonstrated superior accuracy and achieved a low RMSE of 0.108 ± 0.068 mm. The study highlighted that optimizing hyperparameters enabled the GRU model to surpass the accuracy of existing models, with an improvement of 25-30% in performance (44).

In contrast to the existing methods, the proposed Time Delay Compensating Motion Estimation Net (TDCMP Net) adopts a unified hybrid architecture that integrates spatiotemporal feature extraction, temporal dependency modeling, and respiratory signal reconstruction within a single end-to-end framework. Furthermore, by jointly learning these complementary components, the proposed approach is designed to robustly handle irregular respiratory patterns and long-range temporal variations commonly observed in clinical respiratory signals.

## 2 Materials and methods

### 2.1 Dataset description

The study utilized PET/CT images from two esteemed medical facilities in Assam, India: The Northeast Cancer Hospital and Research Institute and Nucleomed Imaging & Diagnostic Centre. Data collection comprising PET and CT series was clinician-supervised to ensure consistency. The study involved only secondary use of de-identified data and did not include any human subject intervention or direct patient participation. Furthermore, all studies were de-identified prior to transfer and patient identifiers such as patient information, history, and annotations were removed to ensure privacy and confidentiality.

The dataset encompasses (i) soft-tissue Hounsfield Units (STHU), (ii) boundary/edge Hounsfield Units (edgeHU), (iii) overall motion in the region of interest (MotionROI), and (iv) estimated range of motion (EROM) per respiratory cycle for each patient. In addition, motion descriptors are expressed along three anatomical axes namely superior-inferior (SI), anterior-posterior (AP), and left-right (LR), with amplitudes reported in millimeters.

We analyzed 1777 patients which yielded approximately 400,000 respiratory signal segments (225 segments per patient: 15 slices x 15 signal variations). To prevent subject leakage, dataset splits were performed patient-wise with 80% for training, and 20% for testing. In addition, random seeds were fixed to ensure reproducibility of all reported results. Moreover, for each patient, 15 representative axial slices covering the tumor extent and adjacent motion-affected regions were selected under clinician supervision, and each respiratory signal segment corresponded to a 120-second acquisition window per bed position.

### 2.2 Critical considerations about the dataset

A combination of practical constraints, regional limitations, and clinical benefits drove the choice of PET/CT over 4DCT. At the time of our research, 4DCT was not readily accessible, considering the resource-constrained setting in a developing country, thereby limiting the choice of modalities. Moreover, beyond the logistical constraints, PET/CT were considered to attain combined functional and anatomical information as it provides insights into tumor metabolism with precise localization. The implementation of dual-modality imaging meets the demand for spatial information across slices and enhances the comprehension of respiratory variations resulting from tumor position changes over time. However, it has to be acknowledged that the respiratory surrogates were derived from HU dynamics and fused PET/CT context, and noting that these are algorithm-level surrogates rather than direct internal fiducials.

Although 4DCT is an advanced technique that can capture the complete spectrum of motion information for both the lung and tumor, irregular breathing patterns can cause fluctuations in baseline shifts. The alterations of baseline respiratory patterns can cause significant modifications in patient anatomy compared to simulated data, resulting in dosimetric errors (45). These dosimetric errors result in misaligning the tumor geometry, leading to suboptimal treatment outcomes (46). Moreover, accurately estimating lung motion concerning the respiratory phase presents challenges associated with image registration issues due to motion artifacts and the considerable interslice thickness in 4DCT. In addition, conventional registration methods treat each phase image in 4DCT independently, which can compromise the temporal coherence across respiratory phases (47).

Despite significant advancements in modern CT scanners focusing on improved speed and resolution, the basic measurements to image larger tissue volumes using multidetector hardware remain density and volume. Density is measured using the HU scale, where air is approximately minus (-)1000 (48). Considering that the lung primarily comprises air and tissue, the density in HU units can directly correlate with the air and tissue content within the imaged region of interest. Moreover, the exact HU units of air and tissue vary over time (49). The edges at tumor-parenchyma interfaces are generally sensitive to motion-induced blur and partial volume effects (50, 51). While single slices may not reveal motion clearly, multi-slice and multi-time sampling exposes cyclic variations at different stages of the respiratory cycle (52, 53).

Considering these findings, we quantified the voxel-wise HU statistics within tumor volumes and at tumor boundaries across slices and time, while also computing per-axis mean amplitudes with time as a fourth dimension. Subsequently, using various slices, the mean values for the tumor boundaries impacted by respiration were computed to assess the variations in tumor positions across the respiratory cycle. The measurement tool and the time-intensity curves in the Medixant RadiAnt DICOM viewer enabled consistent motion evaluation across multiple slices of various patients (54). The present dataset and surrogates support algorithmic validation of respiratory motion prediction. In addition, the direct physical calibration to implanted markers or fluoroscopy, and dosimetric consequence analysis are out of scope in this study and planned in the follow-up work.

### 2.3 Image processing

**Figure 1:**
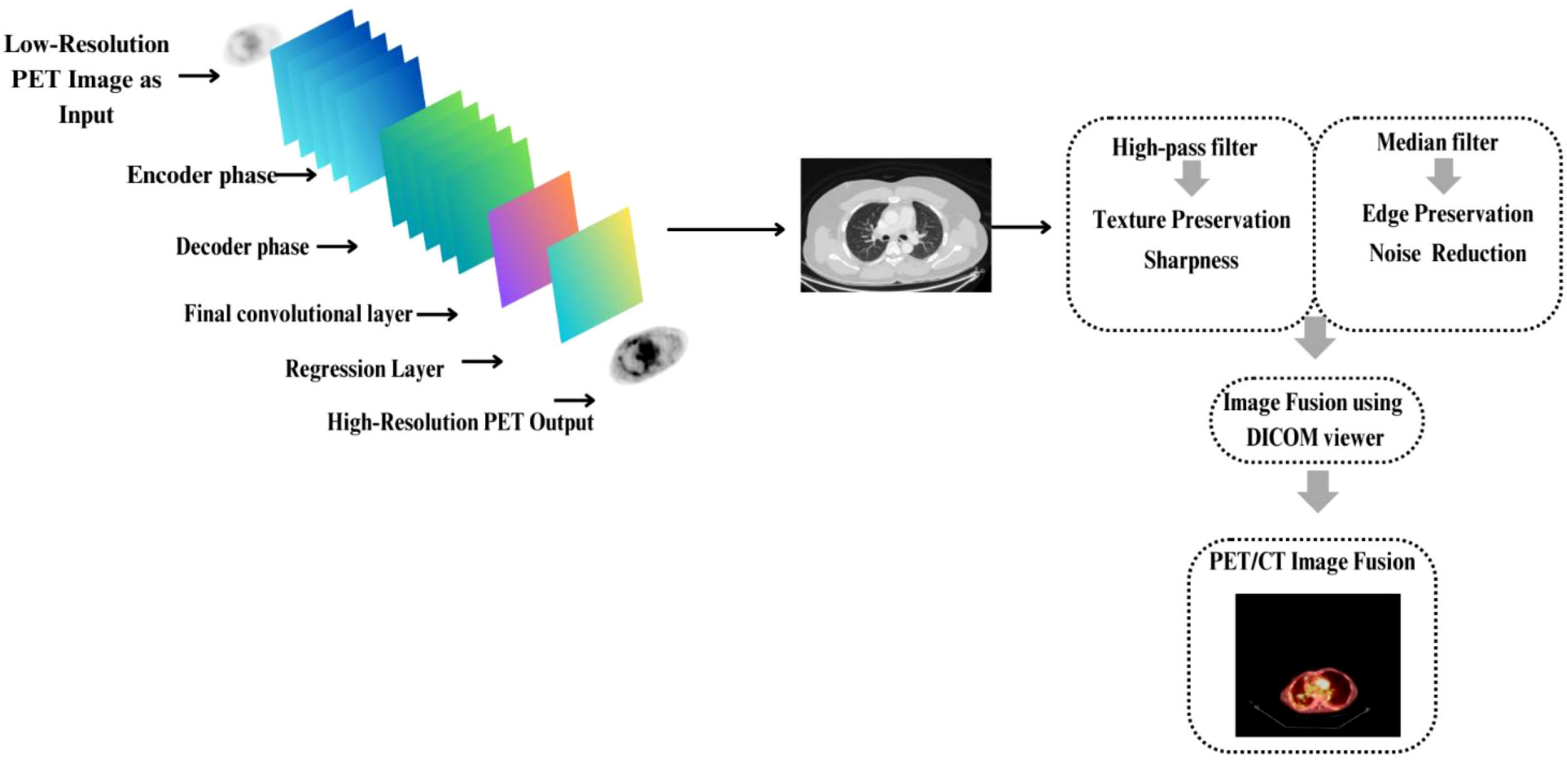
PET/CT preprocessing framework, including PET super-resolution, CT denoising, and multimodal fusion for downstream respiratory motion analysis.

The PET images were processed using a preprocessing framework based on a super-resolution approach to enhance image quality prior to multimodal fusion with CT images. Super-resolution was applied to compensate for the inherent spatial resolution limitations of low-dose PET imaging and to improve structural fidelity for subsequent motion analysis. **Figure 1** depicts the entire preprocessing framework.

U-Net is a prominent deep-learning architecture developed by Ronneberger et al. for biomedical image segmentation and has demonstrated its effectiveness across a diverse range of biomedical imaging tasks (55). The U-Net was adopted and fine-tuned using transfer learning to perform image-to-image regression. Furthermore, the network learns a direct mapping from low-resolution PET images to enhanced resolution outputs by prediction continuous valued intensity images from the input data. U-Net architecture was selected due to its encoder-decoder structure with skip connections, which enables effective capture of spatial hierarchies while preserving both high-level contextual information and fine-grained structural details.

The generalized equation for image-to-image-regression is expressed as:

(1) $$\hat{\text{x}}={\text{f}}_{\text{θ}}(\text{x})$$

In Equation 1, x denotes the input low-resolution PET image, $\hat{\text{x}}$ represents the reconstructed high-resolution output, and *f_θ_* is the learned mapping parameterized by network weights _θ._ However, unlike the traditional iterative super-resolution formulations that rely on explicit degradation and inversion operators, the proposed approach directly learns the super-resolution transformation in a supervised manner. This approach allows to introduce hand-crafted operators and ensures stable reconstruction performance for downstream multimodal fusion.

The objective of the super-resolution process was to recover clinically relevant structural details that may be obscured in low-dose PET images. These details are crucial for accurate fusion with CT data as it improves the spatial consistency for subsequent respiratory motion analysis. While specific analytical formulations for deep-learning based super resolution in medical imaging are not universally standardized, a generalized learning-based approach was adopted by considering insights from existing studies (56, 57).

Furthermore, the quality of reconstructed PET images was quantitatively evaluated using the Peak Signal-to-Noise Ratio (PSNR), which measures the relative strength of the reconstructed signal compared to noise. A higher PSNR value indicates superior noise reduction and improved image quality in the reconstructed image (58). The PSNR is the maximum value of a signal and the strength of noise disrupting the image. In the context of the recovered PET images, the PSNR value obtained is indicated by highlighting the scores of the first ten images. For benchmarking purposes, the scores were compared with the PSNR values obtained by the original PET images acquired from the clinical setting. **Table 1** depicts the achieved PSNR scores.

**Table 1:** Comparison of PSNR values between original and reconstructed PET images.

PSNR values of original images obtained from clinical setting (visible poor quality considered)	PSNR of reconstructed PET image
16.09	20.66
18.25	25.77
15.45	23.24
19.77	28.95
18.92	25.45
21.65	29.70
15.81	22.47
16.15	20.34
19.97	24.72
20.52	29.95

**Figure 2:**
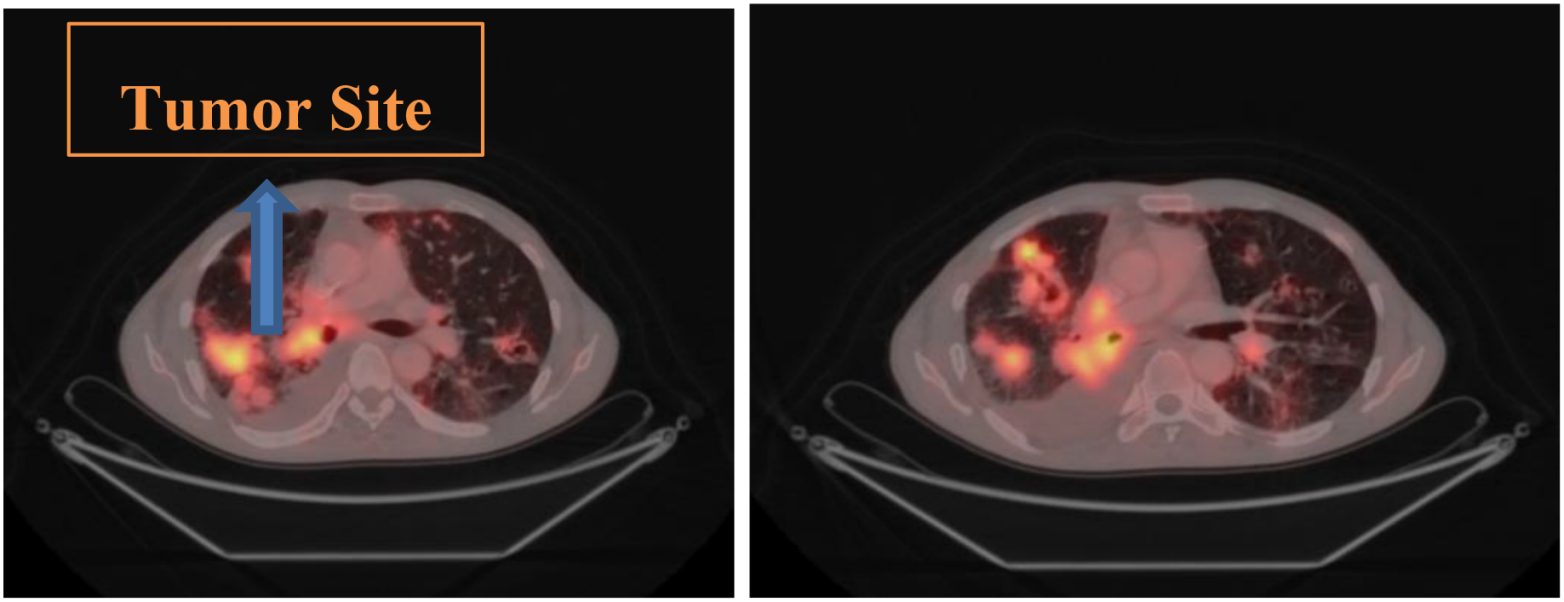
Slice-based tumor localization using fused PET/CT images, illustrating spatial variation across axial slices used for respiratory motion surrogate extraction.

However, CT images are often affected by noise. Therefore, these images were In addition preprocessed to mitigate noise artifacts using a combination of median filtering and high-pass filtering. The median filter reduces random noise while preserving the edges, whereas the high-pass filter enhances structural sharpness and texture contrast. The Medixant RadiAnt DICOM viewer was also used to fuse PET/CT images to assess tumor volumes and positional changes across slices. This information provided the spatial information required for the study. **Figure 2** illustrates the detailed analysis of spatial variations specific to each patient slice, which helps explain the changes in uptake levels and respiratory motion.

#### 2.3.1 Quantitative feature extraction

**Figure 3:**
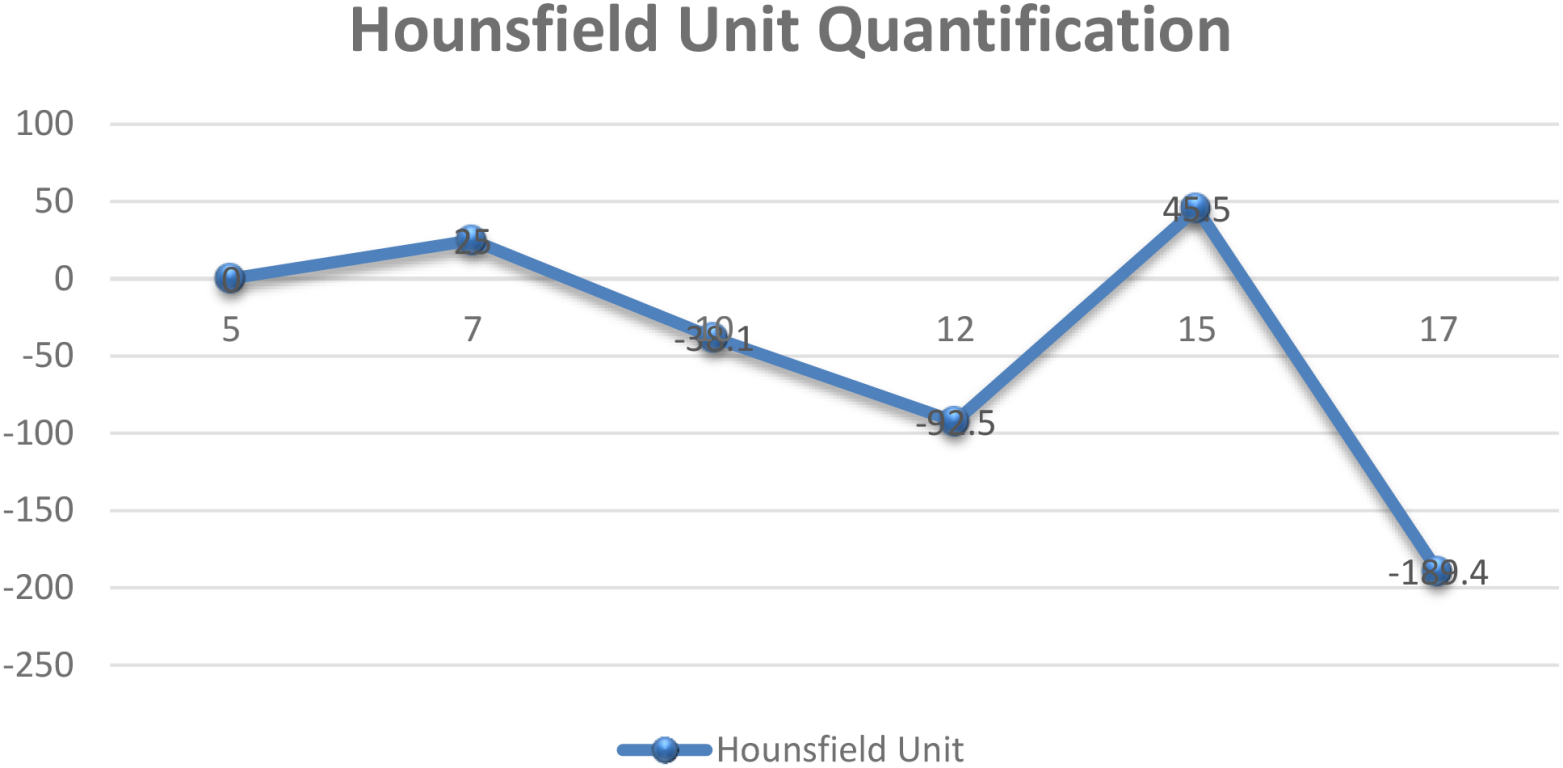
Quantification of Hounsfield Unit (HU) variations across slices using time–intensity curves, illustrating respiratory-induced signal fluctuations.

Quantitative HU information and time-intensity curves were used to capture variations over time intervals which contributed to the temporal dimension of the dataset. **Figure 3** depicts the data that represents the quantitative HU information acquired from a sample of a patient chosen at random, utilizing the time-intensity curve.

**Figure 4:**
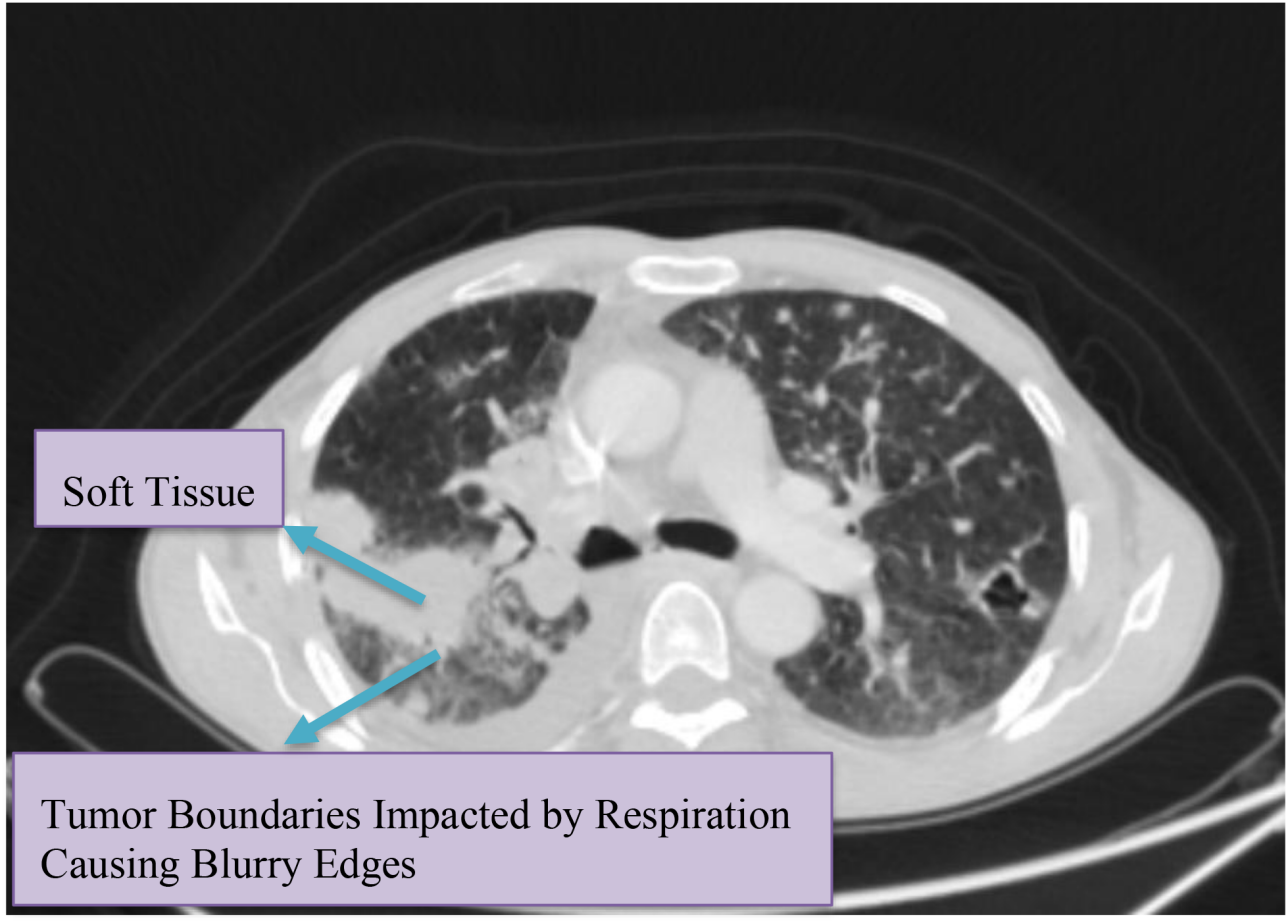
HU sampling across tumor volumes and boundaries over multiple CT slices to capture respiration-related density variations.

The y-axis in **Figure 3** depicts Hounsfield Unit (HU) values derived from time–intensity curves, representing respiratory-induced motion variations of lung tumors across slices, while the x-axis corresponds to slice numbers. Motion within the lung region of interest was quantified using the DICOM viewer’s measurement tool to assess three-dimensional displacement and irregular breathing patterns. Moreover, the differences in HU values between tumor volumes and adjacent tissues were analyzed to estimate motion ranges across respiratory phases, from end-inhalation to end-exhalation. All measurements were reviewed by clinicians to confirm dataset accuracy. When integrated with time–intensity curves, the quantified HU data formed the raw respiratory signals used for model training. **Figure 4** further illustrates the variations in soft-tissue HU values and tumor boundaries influenced by respiratory motion.

### 2.4 Respiratory signal acquisition from raw signals

**Figure 5:**
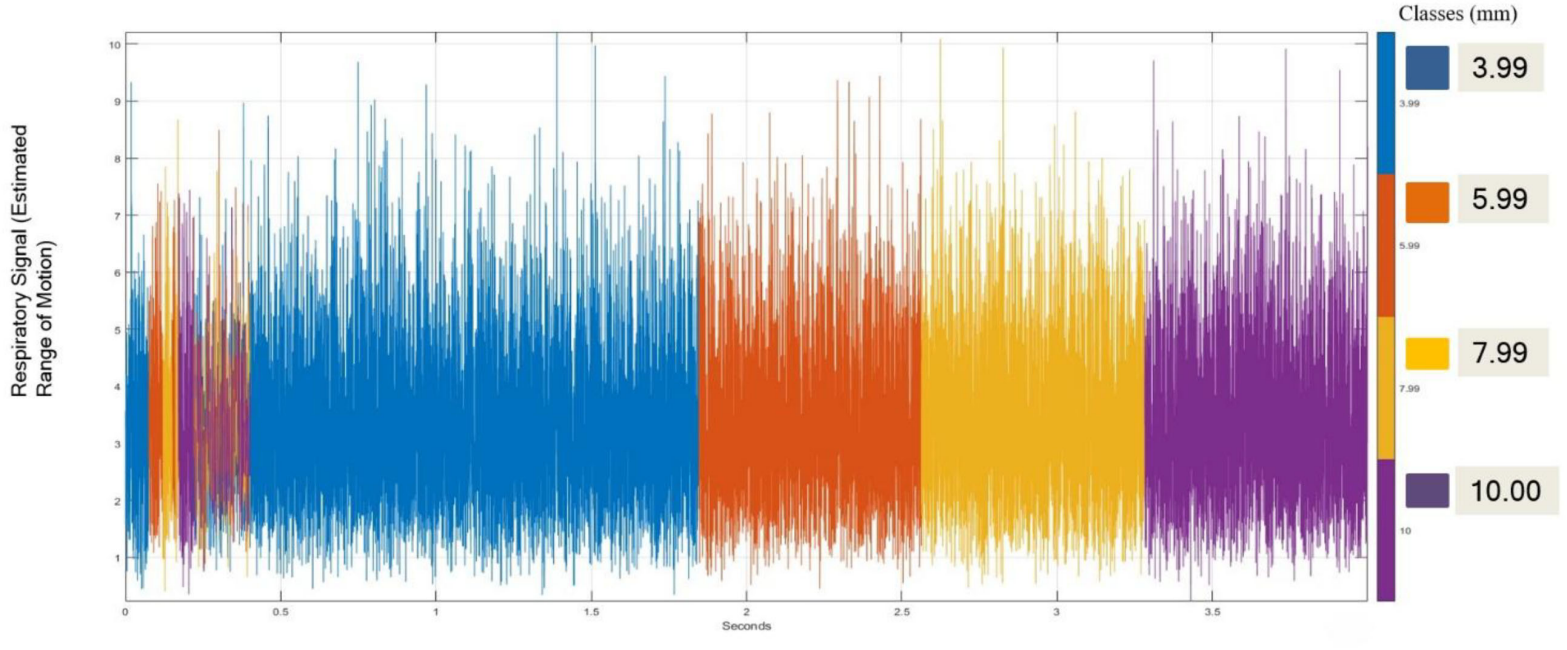
Representative raw respiratory surrogate signals derived from PET/CT HU dynamics, color-coded according to predefined motion-amplitude ranges.

The primary objective was to extract respiratory signal information for each patient across the full axial range which is from the skull base to the mid-thigh in accordance with established clinical imaging guidelines (59). Respiratory signals were derived from variations in Hounsfield Unit (HU) values corresponding to tumor volumes and boundaries, as well as from time–intensity curves analyzed using the RadiAnt DICOM viewer. The acquisition protocols such as the step-and-shoot and continuous bed motion (CBM) methods were considered to determine the exact threshold. The step-and-shoot technique acquires images sequentially by pausing at each bed position, whereas CBM enables continuous scanning by adjusting bed speed and axial range. This method involves the bed moving and pausing at specific intervals to capture images throughout the body. The scanning is determined by numerous factors, such as the number of minutes per bed position and the total number of positions required to cover the axial range (60). A new technology called the continuous bed position (CBM) is implemented to determine scan times by adjusting bed speed and axial ranges (61). Therefore, based on these guidelines, respiratory-signal windows of 120 seconds per bed position were defined for each patient (62). The raw data were processed in the MATLAB Signal Analyzer environment. Since the dataset encompassed nearly 400,000 signal entries, only representative samples are shown for visualization. The processed respiratory signals were color-coded to classify motion ranges used in subsequent modeling. **Figure 5** illustrates these raw signals.

Four motion-amplitude ranges (bins) were defined based on HU-derived motion amplitudes namely 0-4mm (Class 1), 4–6 mm (Class 2), 6–8 mm (Class 3), and ≥8 mm (Class 4). While Class 4 represents high-amplitude respiratory motion, an additional threshold was defined to identify clinically concerning extreme motion. Specifically, motion amplitudes ≥9 mm were flagged as excessive motion, reflecting deviations beyond commonly reported planning margins and gating tolerances. However, for ease of reporting, each class is referenced by a representative value corresponding approximately to the class midpoint (3.99 mm, 5.99 mm, 7.99 mm, and 10.00 mm, respectively). These bins were used consistently throughout training, evaluation, and reporting. In addition, these thresholds were also established from clinical literature and expert oncologist input, which indicate that over half of lung tumors exhibit motion exceeding 5 mm, while those located near the diaphragm may vary between 3 mm and 40 mm (63). Previous studies using the maximum-intensity-projection imaging across full respiratory cycles recommend refining tumor delineation through visual validation over multiple breathing phases to ensure accurate volume definition. The Internal Target Volume (ITV) thereby accounts for physiological respiratory motion, encompassing the Internal Gross Target Volume (IGTV) with an additional margin, typically 8 mm, to include microscopic spread (64). Moreover, clinical literature emphasize a 5 mm gating margin is commonly adopted to mitigate uncertainties, and lung-tumor motion generally ranges between 10 and 15 mm during free breathing, though breath-hold techniques aim to restrict displacement within 5 mm (7, 65).

All the measurements were recorded to two decimal places to capture the physiological variations and to enable the predictive model to generalize across diverse motion profiles. These refined motion ranges were used to train the model for robust characterization of patient-specific tumor motion.

#### 2.4.1 Quantitative feature extraction

Respiratory signals frequently encounter distortions and irregular peak deviations caused by noise and undesired artifacts. In lung cancer imaging, signal inconsistencies may arise due to bed repositioning, variations in multileaf collimator timing, or physiological changes during acquisition. High-frequency noise from amplifiers and motion artifacts, as well as low-frequency fluctuations caused by coughing or breathing irregularities can also degrade signal quality and change the breathing patterns. Moreover, inconsistent breath-hold techniques can disrupt respiratory patterns during simulation and treatment, potentially causing dosimetric and geometric uncertainties (13). In addition, gated radiotherapy can result in incomplete respiratory curves, particularly when the respiratory amplitude lies within the 2–3 mm range. Likewise, surrogate-based signals may become unreliable during prolonged treatment sessions (20).Although respiratory signals usually exhibit non-stationary behavior, they can demonstrate stationary characteristics during controlled breath-hold techniques during data acquisition. Therefore, to effectively handle these challenges, Discrete Wavelet Transform (DWT) was applied, which is well-suited for analyzing non-stationary biomedical signals and for suppressing Gaussian noise. The DWT decomposes the signal into localized frequency components, reducing redundancy and capturing temporal-spatial variations.

The general mathematical formulation of DWT is expressed in Equation 2 which is:

(2) $$W(l,\;m)=\sum_{n=0}^{N-1}\;x(n)\psi_{\left(l,m\right)}(n)$$

Here, *W (l, m)* denotes the wavelet coefficient at scale l and temporal position m. The input respiratory signal is represented by x(n), where n indexes the discrete time samples and N denote the signal length. The function $\psi_{\left(l,m\right)}(n)$ represents the wavelet basis obtained by scaling and translating a mother wavelet, which enables localized time-frequency analysis of the signal.

**Figure 6:**
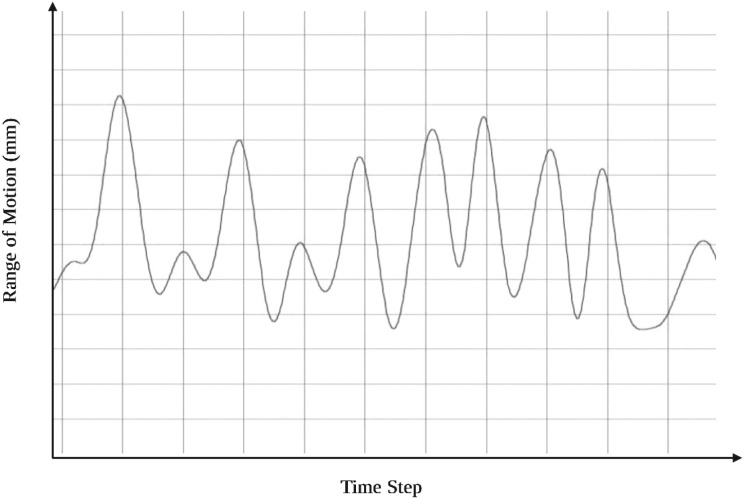
Extracted and denoised respiratory waveforms corresponding to individual breathing cycles after wavelet-based preprocessing.

The DWT decomposes respiratory signals into multiresolution components which allows the separation of slow-varying trends and rapid fluctuations. High-frequency wavelet coefficients capture abrupt signal variations and suppress low-frequency drift, while low-frequency coefficients preserve the respiratory pattern and attenuate broadband noise. This multiresolution decomposition produces clean, denoised respiratory cycles suitable for subsequent modeling. **Figure 6** illustrates representative extracted respiratory waveforms corresponding to individual breathing cycles.

### 2.5 Hybrid network architecture for respiratory motion prediction

**Figure 7:**
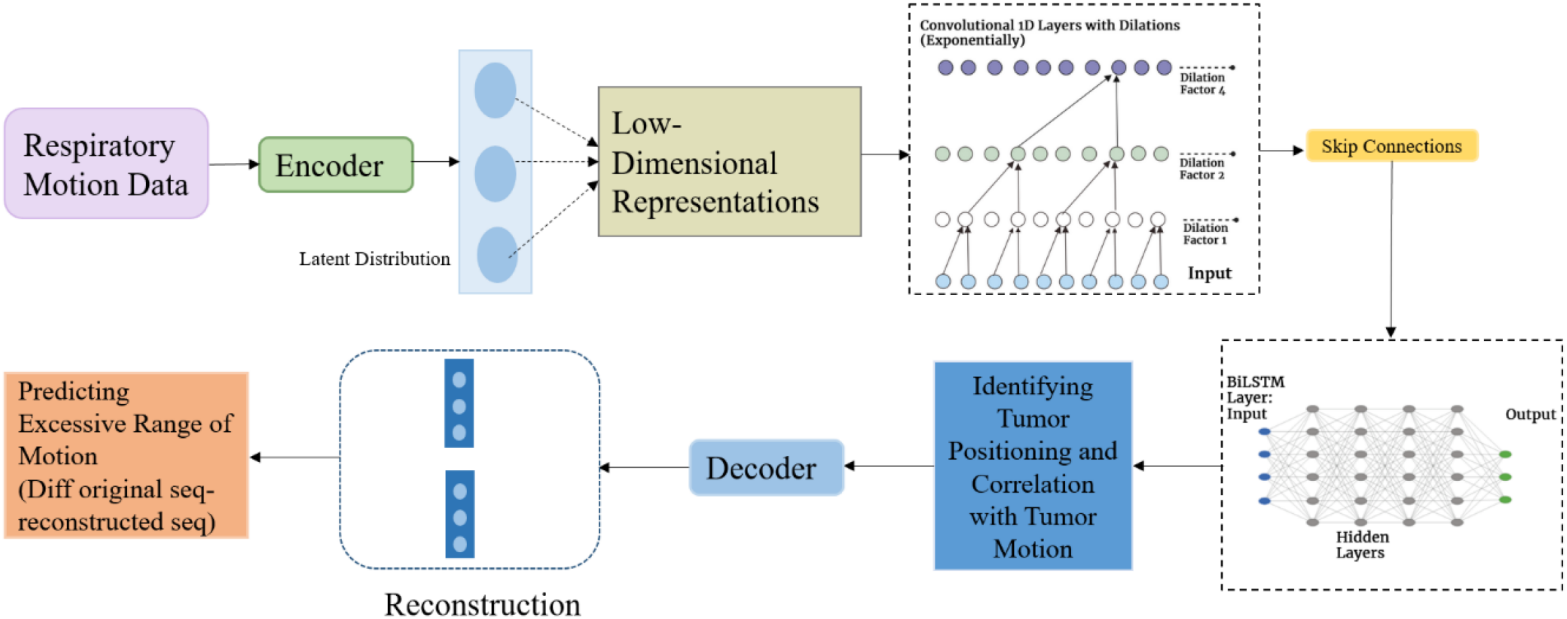
Proposed hybrid deep generative model for respiratory motion prediction.

A hybrid deep learning architecture called the Time Delay Compensating Motion Estimation Net (TDCMP) was developed to predict respiratory-induced tumor motion. In this study, the term hybrid refers to the integration of heterogeneous deep learning components within a single end-to-end architecture. The dilated convolutional layers perform spatiotemporal feature extraction, bidirectional LSTM layers focus on temporal dependencies, and an embedded autoencoder module enables respiratory signal reconstruction. Moreover, these components function as complementary layers or modules rather than independent networks. This enables identification of patients with excessive motion variations that exceed clinical thresholds defined for treatment planning. Furthermore, the model establishes the correlation between tumor position and its three-dimensional motion range, thereby improving delineation accuracy across respiratory phases. In radiotherapy systems where timing is critical, the TDCMP Net facilitates real-time motion prediction, enabling precise gated-beam delivery and minimizing radiation exposure to healthy tissues. **Figure 7** represents the proposed hybrid deep generative model.

The dataset comprising 400,000 respiratory signals was converted into a time-series dataset. However, to ensure computational efficiency and mitigate redundancy, the model generates low-dimensional representations that retain essential motion features while suppressing noise. This dimensionality reduction enhances both training speed and prediction accuracy.

The encoder extracts temporal and spatial features of respiratory signals, and transforming them into compact latent representations. Similarly, the dilated CNN layers capture spatial dependencies that reflect dynamic, non-stationary breathing patterns with rapid temporal fluctuations. Sliding kernels were used to enable the dilated layers to effectively capture long-range dependencies without increasing kernel parameters. This approach offers a larger receptive field, efficient computation, preserved temporal order, and reduced memory usage. Furthermore, layer normalization standardizes intermediate distributions for stable training, while spatial dropout mitigates overfitting by dropping entire feature maps rather than individual neurons, which further enhances generalization. The dilated convolution operation as indicated in Equation 3 is mathematically defined as:

(3) $$\left(F{*}_{d}k)(p)\;=\;\sum_{i}F(p-d.i)k(i\right)$$

where *F* denotes the input feature map and ***k*** represents the convolutional kernel. The operator *_d_ indicates dilated convolution with dilation factor *d*, which controls the spacing between kernel elements. The index *i* enumerates the kernel positions, while *p* denotes the output position. Dilated convolution expands the effective receptive field without increasing the number of learnable parameters, which enables efficient long-range temporal dependencies in respiratory signals. In this formulation, setting ***d=1*** recovers the standard discrete convolution operation.

Subsequently, the BiLSTM layer utilizes spatially encoded features extracted by the dilated CNN to learn temporal dependencies and classify tumor motion ranges throughout each patient’s respiratory cycle. Its bidirectional structure enables the network to capture information from both preceding and succeeding time steps, which is essential for characterizing irregular breathing patterns. The mathematical formulation of the BiLSTM are presented in Equations 4–6 and are defined as follows:

Forward LSTM:

(4) $$y_{t}^{forward}=\text{LSTM}(x_{t},h_{t-1})$$

Backward LSTM:

(5) $$y_{t}^{backward}=\text{LSTM}(x_{t},h_{t+1})$$

Concatenation:

(6) $$y_{t}=y_{t}^{forward},\;y_{t}^{backward}$$

Here, $y_{t}^{forward}$ denotes the output at time step t, where $x_{t}$ represents the input feature vector at time *t*, and $h_{t-1}$ indicates the hidden state from the previous time step *(t-1).*

Similarly, $y_{t}^{backward}$ represents the output of the backward LSTM which processes the input sequence in reverse order, using $x_{t}\;$ as the input data and $h_{t+1}\;$ as the hidden state from the succeeding time step *(t+1).*

Equation 6 highlights the concatenation of the forward and backward LSTM outputs, producing a unified representation $y_{t}$ that captures bidirectional temporal context for the BiLSTM layer.

Given the incorporation of irregular motion patterns in the data, ensuring prediction accuracy is paramount. Therefore, an autoencoder module is strategically integrated within the network. The encoder processes the input respiratory signal by extracting motion-related features and compressing the signal into a lower-dimensional latent representation. The encoder operation is mathematically defined by Equations 7, 8 and is expressed as follows:

(7) $$\text{z}={\text{L}}_{\;\phi }(\text{x})=\text{f}(\text{Wx}+\text{b})$$

where $L_{\;\phi }\;$ denotes the encoder function that maps the input signal x to a latent representation z using learnable weights W and bias b, parameterized by ϕ.

The decoder reconstructs the respiratory signal from the latent representation and is defined as:

(8) $$\hat{\text{x}}={\text{M}}_{\text{θ}}\;(\text{z})=\hat{\text{f}}(\hat{\text{W}}\text{z}+\hat{\text{b}})$$

**Figure 8:**
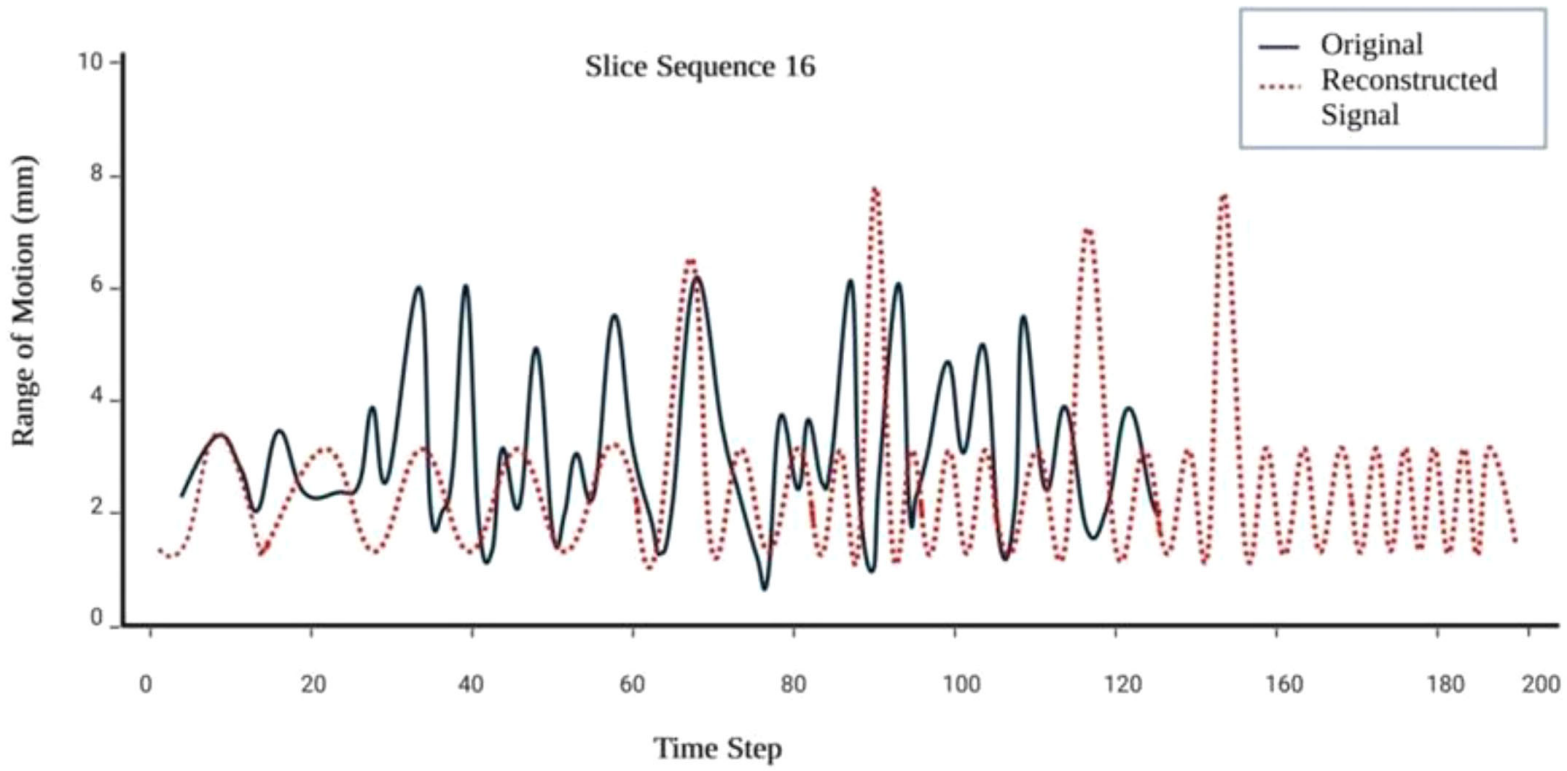
Reconstructed respiratory signals illustrating algorithmic recovery of physiologically coherent breathing cycles from PET/CT-derived respiratory surrogates.

Here, ${\text{M}}_{\text{θ}}$ denotes the decoder function parameterized by θ, which reconstructs the signal $\hat{\text{x}}$ from the encoded representation z. The decoder operates by integrating latent features learned from the dilated CNN and BiLSTM layers, enabling reconstruction of physiologically coherent respiratory waveforms. This reconstruction supports identification of inconsistencies and excessive motion patterns across respiratory cycles. Furthermore, the autoencoder is optimized by minimizing the mean absolute error (MAE) between the original respiratory signal and its reconstructed counterpart to ensure preservation of motion characteristics. **Figure 8** depicts the reconstructed signals corresponding to individual breathing cycles across multiple patient slices.

**Figure 9:**
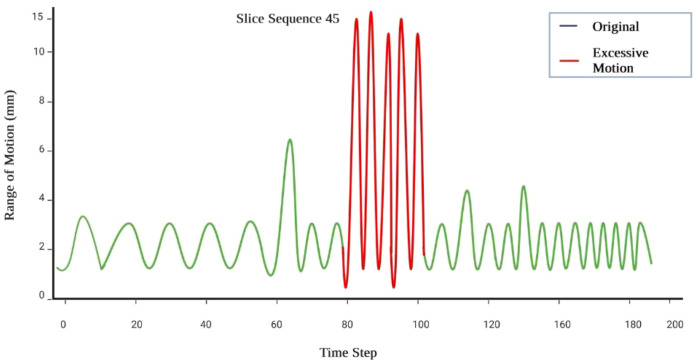
Identification of excessive respiratory motion segments exceeding predefined thresholds, highlighting algorithm-level motion irregularities.

The predicted motion information provides a quantitative framework for correlating tumor position with motion range in three dimensions, improving delineation of clinical margins during planning, simulation, and beam delivery. These predictive insights allow synchronization of radiation gating with patient-specific motion patterns, thereby ensuring precise tumor targeting while minimizing dose exposure to surrounding healthy tissues. **Figure 9** illustrates the outcomes of the excessive motion prediction.

### 2.6 Evaluation metrics and validation protocol

Model performance was evaluated using classification and regression metrics including accuracy, precision, recall, F1-score, area under the ROC curve (AUC), root mean square error (RMSE), and mean absolute error (MAE). All reported results correspond to held-out test data. Statistical uncertainty estimation, such as confidence intervals or hypothesis testing, was not performed in the present study, as the primary objective was algorithmic validation rather than inferential clinical testing. Therefore, formal statistical reliability analysis will be incorporated in future work involving prospective clinical validation and physical-space calibration.

## 3 Results

**Figure 10:**
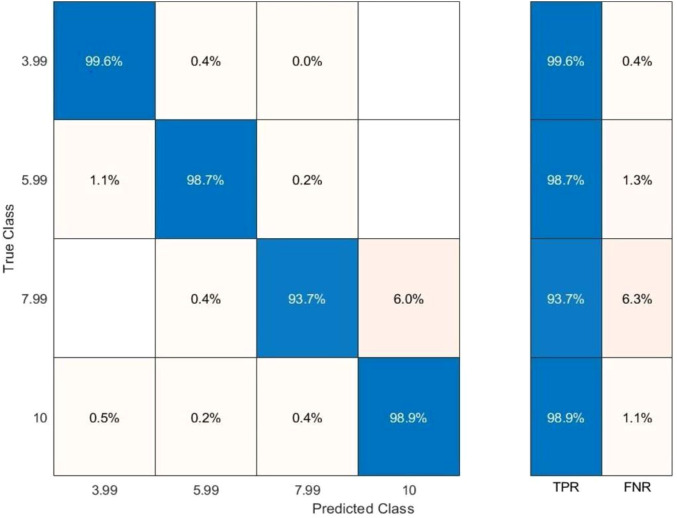
Confusion matrix illustrating motion-range classification performance on the training dataset across four clinically relevant amplitude ranges.

The respiratory signals were categorized into four motion-amplitude ranges: 0–4 mm (Class 1), 4–6 mm (Class 2), 6–8 mm (Class 3), and ≥8 mm (Class 4) (reported using representative values of 3.99 mm, 5.99 mm, 7.99 mm, and 10.00 mm, respectively). **Figure 10** illustrates the classification accuracy of the proposed model on the training dataset.

**Figure 11:**
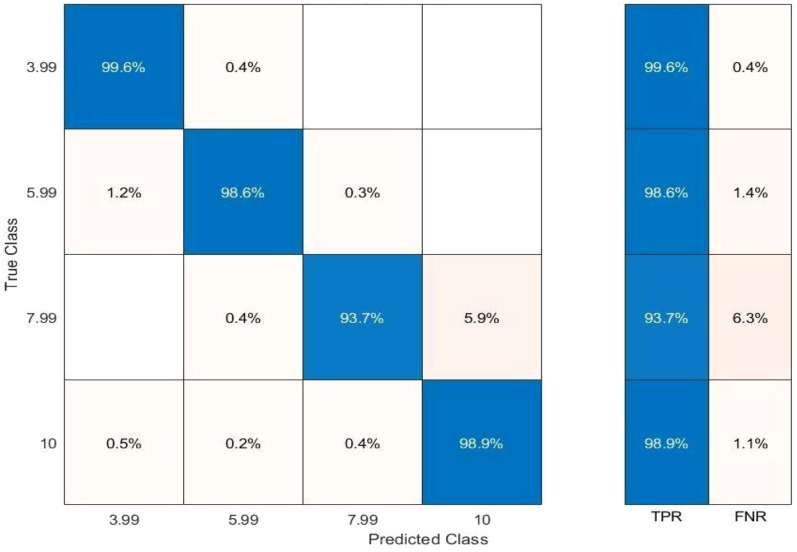
Confusion matrix illustrating motion-range classification performance on the test dataset across four clinically relevant amplitude ranges.

The confusion matrix demonstrates high reliability, achieving true positive rates of 99.6% (3.99 mm), 98.7% (5.99 mm), 93.7% (7.99 mm), and 98.8% (10 mm). False-negative rates were negligible across all classes, confirming robust classification. Based on the outcomes, it can be inferred that the classification accuracy is significant and highly reliable. The results reveal a true positive rate of 99.6% for class 1 (3.99 mm), followed by a true positive rate of 98.7% for class 2 (5.99 mm), 93.7% for class 3 (7.99 mm), and 98.8% for class 4 (10 mm). Similarly, the classification results for the test data are indicated in **Figure 11**.

**Figure 12:**
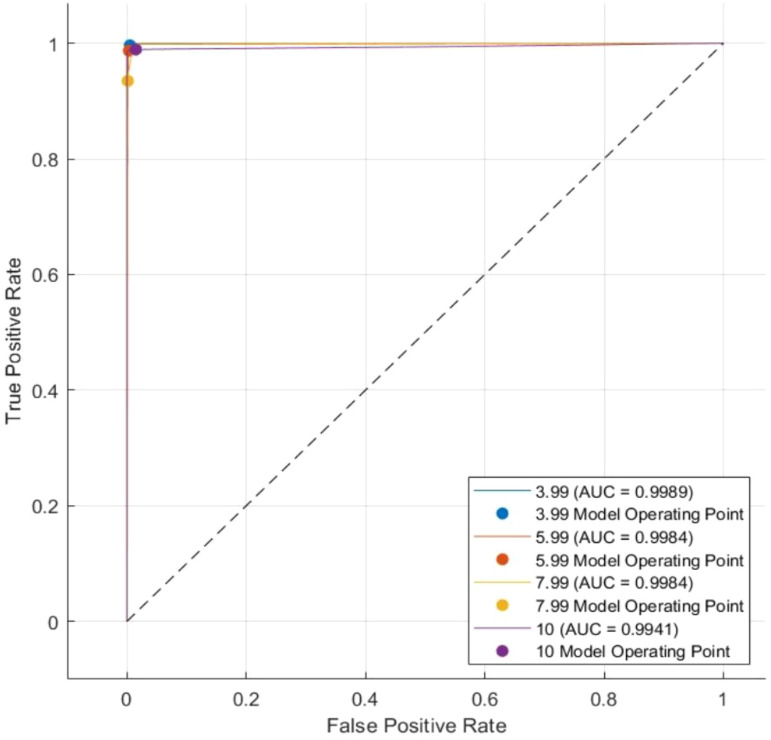
AUC curve for the train set.

The results indicate that the proposed model retains its accuracy when applied to the test data, with slight variations in class 2 and a significant improvement in class 4. The ROC–AUC analysis further validates classification performance. An effective classifier has a TPR score of 1 and an FPR score of 0 on the ROC curve. The model attained an AUC score of 0.9989 for class 1 (3.99 mm), 0.9984 for class 2 (5.99 mm), 0.9984 for class 3 (7.9 mm), and 0.9941 for class 4 (10 mm) for the training data as depicted in **Figure 12**.

**Figure 13:**
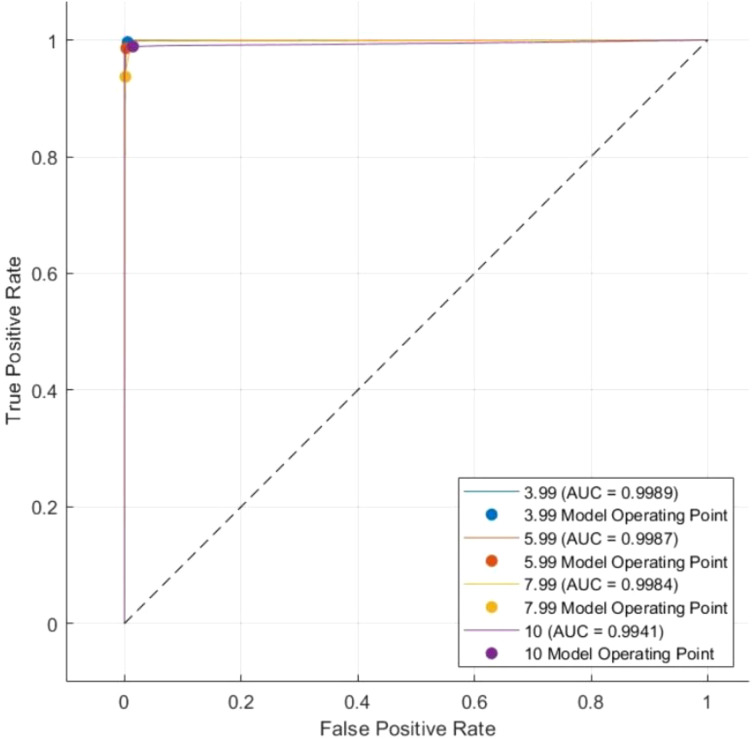
AUC curve for the test set.

In addition, for the test set, the model’s ability to differentiate between the classes and accurately classify the ranges of motion was exceptional, as demonstrated by the remarkably high AUC scores of 0.9989 for class 1 (3.99 mm), 0.9987 for class 2 (5.99 mm), 0.9984 for class 3 (7.9 mm), and 0.9941 for class 4 (10 mm). These AUC scores indicate that the proposed classification approach is robust and effective, as a higher AUC score implies better discrimination between positive and negative instances. **Figure 13** depicts the AUC curve for the test set.

While accuracy is an important metric to evaluate the model’s performance, it is insufficient for imbalanced classes. To evaluate the model’s performance on imbalanced classes, F1 scores were computed for each range. Precision, recall, and F1-score were also computed for each motion range to assess robustness under imbalanced conditions.

Precision and recall were calculated using the standard formulations, as defined in Equation 9:

(9) $$\text{Precision}=\frac{TP}{TP+FP},\text{Recall}=\frac{TP}{TP+FN}$$

where TP, FP, and FN denote true positives, false positives, and false negatives respectively. The F1-score, defined as the harmonic mean of precision and recall, provides a balanced measure of the model’s ability to minimize false classifications. This is defined in Equation 10.

(10) $$F1=2\times \frac{\text{Precision}\times \text{Recall}}{\text{Precision}+\text{Recall}}$$

The consistently high F1-scores for both training and test data confirm the model’s strong ability to classify respiratory signal sequences across all motion ranges. **Table 2** summarize the F1-scores for each range, highlighting the model’s precision and reliability.

**Table 2:** F1 scores for each class on training and test data.

Estimated range of motion (mm)	F1 score (training data)	F1 score (test data)
3.99 mm	0.9961	0.9962
5.99 mm	0.9871	0.9861
7.99 mm	0.9389	0.9386
10.00 mm	0.9890	0.9889

## 4 Comparative analysis

The robustness and performance of the proposed model were evaluated through a comparative analysis. In radiotherapy, motion prediction systems are required to compensate for system latency to ensure accurate tumor tracking and precise dose delivery. Existing research have shown that maintaining tracking latency below 150 milliseconds allows for narrower motion margins compared to conventional gating techniques as it facilitates precise localization of the clinical target volume’s center of mass (66). Furthermore, proton therapy systems often face limitations as beam activation and deactivation latencies can exceed 200 milliseconds, especially when breathing cycles last up to 3–4 seconds (67). However, in advanced radiotherapy systems, the latency can span from 50 to 500 ms, which directly influences synchronization and treatment precision (68). Therefore, considering the findings related to latency considerations in radiotherapy systems, the root mean square error (RMSE) for the three dimensions was calculated for different directions: superior-inferior, anterior-posterior, and left-right. **Table 3** outlines the RMSE scores of the proposed model in three-dimensional space.

**Table 3:** RMSE scores in three-dimensions.

Directions	RMSE
Anterior-posterior	0.0092
Left-right	0.0115
Superior-inferior	0.0127

Reference target positions were derived from PET/CT-based spatiotemporal surrogate information, enabling consistent estimation of tumor localization across respiratory phases. These surrogate-derived references serve as a consistent algorithmic benchmark rather than direct physical ground truth. The extracted spatiotemporal data were analyzed through time–intensity curves representing tumor motion and subsequently transformed into time-series data for predictive modeling. However, it was also noted that uncertainties in determining the exact target position mainly arise from imaging noise, motion artifacts, and inherent modality limitations. Therefore, to mitigate these factors, appropriate image processing and filtering techniques were applied to minimize localization errors. The temporal evolution of tumor position over increasing simulation times was also examined, which revealed a gradual increase in positional deviation as simulation time progressed. This behavior was quantitatively evaluated using root mean square error (RMSE) across different time intervals, which accounted for variations and positional latency factors that may arise during radiotherapy. The computed RMSE values demonstrated minimal average deviation between predicted and actual tumor positions, supporting the model’s precision and stability under realistic clinical conditions. The close agreement between predicted and ground-truth positions confirms the model’s ability to accurately predict respiratory-induced tumor motion across multiple dimensions.

To facilitate comparative analysis, relevant studies in similar research domains were considered, including those by Shi et al. (EMD-SENET-TCN), Wang et al. (Deep BiLSTM), and Yu et al. (BI-GRU) (69). These references were utilized to evaluate the model’s performance concerning existing studies, as shown in **Table 4**.

**Table 4:** RMSE scores in three-dimensions.

Latency range (ms)	EMD-SENET-TCN	Deep BiLSTM	Bidirectional GRU (top five scores of different patients)	Proposed model (TDCMP)
40-50	0.0225	0.0191	0.0841	0.0092
100-150	0.0315	0.0274	0.1707	0.0115
200-300	0.0537	0.0342	0.0883	0.0127
400-500	0.0689	0.0969	0.1335	0.0205

The RMSE results indicate minimal average deviation between the predicted and actual tumor positions. The model’s predictions remained consistently close to the true target locations, sustaining low error rates even across varying latency ranges. These outcomes highlight the model’s precision, robustness, and reliability in capturing respiratory-induced motion.

**Figure 14:**
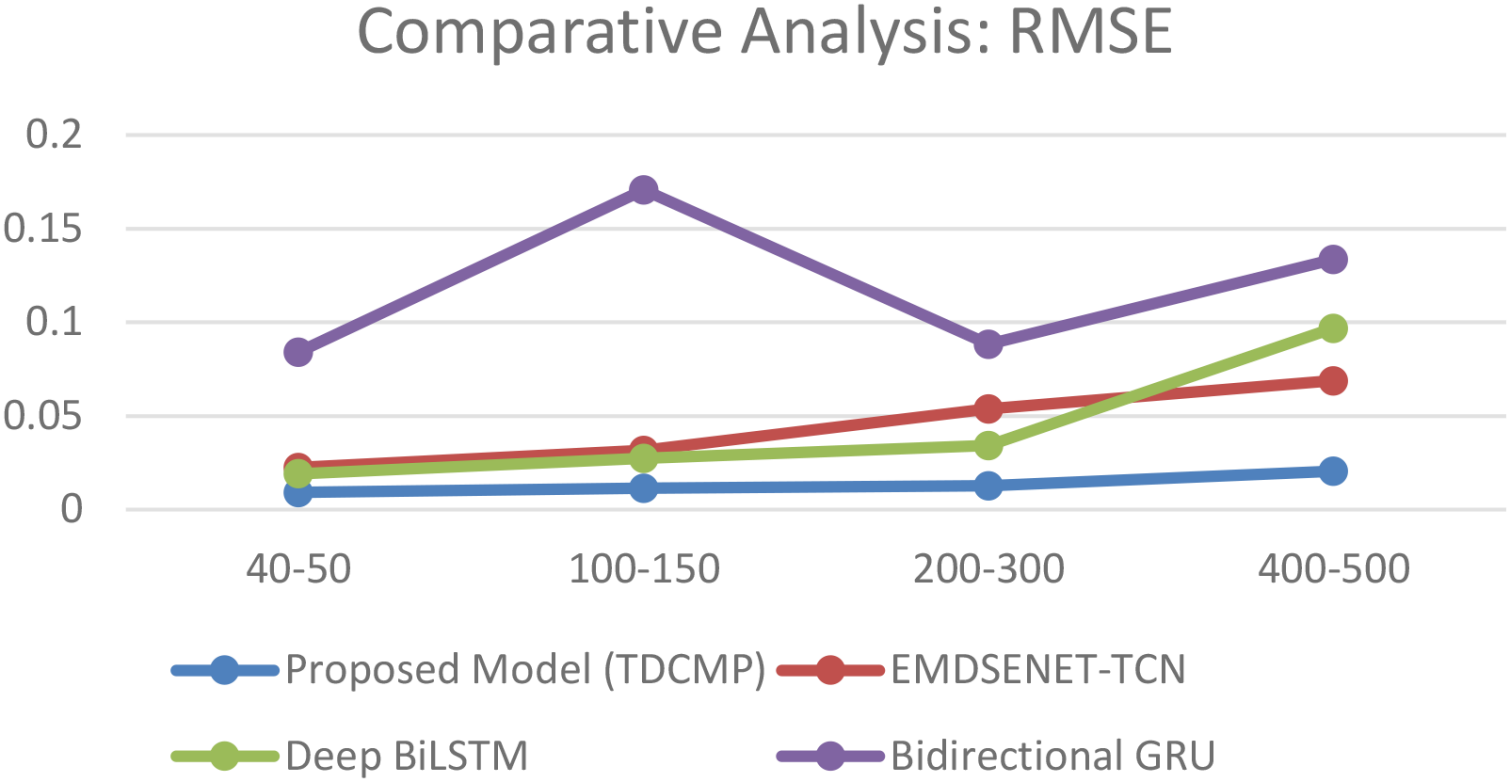
Performance evaluation of the TDCMP Net.

The comparative results reported for existing methods were obtained from their respective published studies, as direct reimplementation on the present dataset was not feasible due to differences in data availability and acquisition protocols. However, the lower RMSE values achieved by the proposed model confirm its accuracy and stability in motion prediction, contributing to improved treatment planning precision and overall radiotherapy effectiveness. The comparative results are illustrated in **Figure 14**.

### 4.1 Mean absolute error for respiratory signal reconstruction

A respiratory signal reconstruction analysis was carried out using data collected from randomly selected patients, each exhibiting noticeable variability in breathing patterns. The signals processed by the preceding layers of the model provided motion range information, which was then reconstructed into representative respiratory curves corresponding to each patient’s unique breathing cycle. Model performance was evaluated using Mean Absolute Error (MAE), where low scores indicated high accuracy in reconstructing the respiratory signals into realistic breathing cycles. The MAE metric is essential for assessing signal reconstruction performance, as it quantifies the absolute difference between predicted and actual values, thereby providing a clear measure of the model’s fidelity in capturing true respiratory behavior.

MAE functions as a reliable performance metric by quantifying the absolute difference between predicted and actual signal values, offering a clear measure of reconstruction accuracy. Its resilience to outliers makes it well-suited for modeling respiratory motion, which often exhibits irregular patterns and abrupt changes. In addition, MAE’s sensitivity to latency variations allows for a comprehensive assessment of the model’s reconstruction capability and motion prediction performance across diverse clinical scenarios. The MAE scores obtained for the reconstruction tasks are summarized in **Table 5**.

**Table 5:** MAE scores for reconstruction.

Latency ranges (ms)	MAE Scores
40-50	0.0075
150-200	0.0100
200-300	0.0110
400-500	0.0180

The findings demonstrate the consistency and accuracy of the proposed model in reconstructing respiratory signal sequences and predicting excessive motion ranges. Motion deviations exceeding the predefined 9 mm threshold were classified as excessive motion. The deep generative network effectively reconstructed respiratory signals and predicted excessive motion up to 500 milliseconds in advance, highlighting its strong predictive capability and potential clinical significance.

## 5 Discussion

This study presents a hybrid deep generative framework for respiratory motion prediction that integrates PET/CT derived information to jointly model spatial and temporal dynamics associated with lung tumor motion. The PET/CT fusion allowed for consistent tumor localization while exploiting temporal variations in voxel intensity to derive respiratory signal surrogates. However, as there is inherent noise and non-stationarity present in such signals, targeted preprocessing was applied to isolate physiologically meaningful respiratory patterns to enable the construction of stable and sequential time-series data suitable for predictive modeling.

The proposed model demonstrates strong and consistent predictive performance across multiple latency horizons and various respiratory signal characteristics and breathing patterns. Most importantly, the results reported in this study are positioned within an algorithmic validation framework, where respiratory motion is inferred from PET/CT derived surrogates rather than directly measured physical motion. This distinction is intentional and reflects the study’s focus on evaluating computational feasibility, temporal stability, and robustness under realistic signal variability. In addition, within this scope, the modeling of latency proved critical, as real-time radiotherapy accuracy depends on anticipating motion across variable system delays. The model’s ability to maintain low prediction error across extended latency ranges highlight its suitability for latency-aware motion compensation scenarios. Furthermore, all reported performance metrics are presented as point estimates to characterize predictive behavior under realistic respiratory variability. Formal statistical uncertainty estimation, such as confidence intervals or hypothesis testing, generally requires prospective clinical validation with physically calibrated motion references and dose-aware evaluation pipelines. As the present work is intentionally focused on algorithmic feasibility and signal-level validation, such statistical analyses are considered outside the scope of this study and are deferred to future clinically integrated evaluations.

In addition, it has been identified that residual motion arising from irregular breathing, baseline shifts, or incomplete compensation of respiratory motion remains a key source of geometric uncertainty in image-guided radiotherapy. Therefore, this model also emphasizes respiratory signal reconstruction and excessive motion identification. The incorporation of a generative reconstruction mechanism enables recovery of physiologically coherent breathing cycles while simultaneously identifying motion amplitudes that exceed nominal clinical thresholds. Hence, such capabilities are essential for characterizing motion variability that may not be fully addressed by conventional gating or tracking approaches and for supporting more informed margin design during treatment planning. Furthermore, a major strength of the proposed architecture lies in its capacity to handle irregular and non-stationary breathing patterns without relying on strict periodic assumptions. In addition, the dataset encompassed both stable and highly variable respiratory behaviors, necessitating a learning framework capable of capturing subtle temporal dependencies over extended sequences.

The integration of dilated convolutional layers, bidirectional temporal modeling, and latent-space regularization allows the model to effectively learn both long-term trends and transient fluctuations, resulting in excellent performance across heterogeneous patient-specific motion profiles. However, from a modeling perspective, careful optimization of learning parameters including regularization strategies, spatial dropout, and normalization was essential for balancing convergence stability and generalization. These design choices contributed to consistent performance across a large-scale dataset while maintaining computational efficiency, which reinforces the framework’s scalability for future translational extensions.

While the present study establishes a strong computational foundation for respiratory motion prediction, direct physical-space calibration and dosimetric impact assessment remain outside its current scope. Future work will focus on staged translational validation, including alignment with established motion reference modalities, latency-aware dose sensitivity analysis, and integration within clinically controlled evaluation settings. The modular and hardware-agnostic design of the proposed framework supports such extensions without modification of the core predictive engine. Collectively, this work provides a robust and extensible algorithmic basis for advancing motion-aware radiotherapy planning and delivery.

## 6 Conclusion

The proposed hybrid model demonstrates strong predictive capability in modeling tumor motion across continuous respiratory cycles by using high-resolution spatiotemporal PET/CT data. The model accurately classifies motion ranges relevant to radiotherapy planning while maintaining consistent precision under varying latency conditions. Furthermore, the effectiveness in processing both stationary and non-stationary respiratory signals, as well as handling irregular breathing patterns, highlights its robustness. The model achieved consistently lower RMSE values, corresponding to an average relative improvement of approximately 29% over representative deep learning–based respiratory motion prediction methods reported in the literature.

Although the proposed system shows high predictive accuracy based on respiratory-signal data, the present study focuses primarily on algorithmic validation. Future work will incorporate spatial calibration in physical units (millimeters) and dosimetric evaluation to directly assess the model’s clinical relevance. Accordingly, this study establishes the computational foundation for developing subsequent clinically interpretable models integrated into radiotherapy planning workflows.

The study also acknowledges certain limitations, including the need for further validation using advanced imaging modalities such as 4DCT and the inclusion of surrogate motion markers. Future research will address these aspects by exploring datasets with external respiratory variations and by implementing patient-specific predictive modeling strategies. These extensions are expected to contribute toward the development of clinically deployable, latency-aware radiotherapy frameworks capable of achieving optimized motion prediction and treatment precision.

## Data Availability

The dataset cannot be shared publicly or upon request as it forms part of an ongoing research and patented work under institutional collaboration. The PET/CT imaging data were obtained from clinical partners under confidentiality agreements, and further use of the anonymized data is currently restricted due to continuing validation studies and intellectual property considerations. Requests to access the datasets should be directed to kaushik.das@res.christuniversity.in.
